# Comparative Genomics Discloses the Uniqueness and the Biosynthetic Potential of the Marine Cyanobacterium *Hyella patelloides*

**DOI:** 10.3389/fmicb.2020.01527

**Published:** 2020-07-07

**Authors:** Ângela Brito, Jorge Vieira, Cristina P. Vieira, Tao Zhu, Pedro N. Leão, Vitor Ramos, Xuefeng Lu, Vitor M. Vasconcelos, Muriel Gugger, Paula Tamagnini

**Affiliations:** ^1^i3S – Instituto de Investigação e Inovação em Saúde, Universidade do Porto, Porto, Portugal; ^2^IBMC – Instituto de Biologia Molecular e Celular, Universidade do Porto, Porto, Portugal; ^3^Key Laboratory of Biofuels, Shandong Provincial Key Laboratory of Synthetic Biology, Qingdao Institute of Bioenergy and Bioprocess Technology, Chinese Academy of Sciences, Qingdao, China; ^4^Interdisciplinary Centre of Marine and Environmental Research (CIIMAR/CIMAR), University of Porto, Matosinhos, Portugal; ^5^Laboratory for Marine Biology and Biotechnology, Qingdao National Laboratory for Marine Science and Technology, Qingdao, China; ^6^Departamento de Biologia, Faculdade de Ciências, Universidade do Porto, Porto, Portugal; ^7^Institut Pasteur, Collection des Cyanobactéries, Paris, France

**Keywords:** biosynthetic gene clusters, cyanobacteria, genome, *Hyella*, natural products

## Abstract

Baeocytous cyanobacteria (Pleurocapsales/Subsection II) can thrive in a wide range of habitats on Earth but, compared to other cyanobacterial lineages, they remain poorly studied at genomic level. In this study, we sequenced the first genome from a member of the *Hyella* genus – *H. patelloides* LEGE 07179, a recently described species isolated from the Portuguese foreshore. This genome is the largest of the thirteen baeocyte-forming cyanobacterial genomes sequenced so far, and diverges from the most closely related strains. Comparative analysis revealed strain-specific genes and horizontal gene transfer events between *H. patelloides* and its closest relatives. Moreover, *H*. *patelloides* genome is distinctive by the number and diversity of natural product biosynthetic gene clusters (BGCs). The majority of these clusters are strain-specific BGCs with a high probability of synthesizing novel natural products. One BGC was identified as being putatively involved in the production of terminal olefin. Our results showed that, *H*. *patelloides* produces hydrocarbon with C_15_ chain length, and synthesizes C_14_, C_16_, and C_18_ fatty acids exceeding 4% of the dry cell weight. Overall, our data contributed to increase the information on baeocytous cyanobacteria, and shed light on *H. patelloides* evolution, phylogeny and natural product biosynthetic potential.

## Introduction

Cyanobacteria are a monophyletic group of Gram negative bacteria with the ability to perform oxygenic photosynthesis and nowadays contribute up to 30% of the annual oxygen production on Earth (Deruyter and Fromme, 2008). They are found in a broad range of habitats (from fresh to salt water, soils and extreme environments) contributing significantly to the global primary production, mainly in nutrient-limited environments (Garcia-Pichel et al., 2003; Flombaum et al., 2013; Díez et al., 2016). In addition, the diazotrophic cyanobacteria constitute the major source of biological nitrogen in the open ocean (Zehr, 2011). Cyanobacteria are also known to produce a wealth of natural products (NPs) with a wide spectrum of noteworthy biological activities such as anticancer, antibacterial, antiviral and antifungal (Nunnery et al., 2010; Sivonen et al., 2010). The importance of cyanobacteria in ecosystems’ equilibrium and the interest in their bioproducts are contributing to enlarge their genomic representation, still largely biased toward marine picocyanobacterial genera. The unicellular strains that divide by multiple fission producing small daughter cells, baeocytes (Subsection II/Pleurocapsales) (Castenholz, 2001), are clearly underrepresented at genomic level. Phylogenetic studies have shown that baeocyte-forming cyanobacteria are distributed in one clade composed by different genera (the major baeocystous clade), plus two separated branches containing *Pleurocapsa* sp. PCC 7327 and *Chroococcidiopsis thermalis* PCC 7203, respectively (Shih et al., 2013; Calteau et al., 2014). Baeocyte-forming strains have an ubiquitous distribution and can be found in terrestrial and desert habitats, in freshwater and marine environments as well as in the intertidal zones (Castenholz, 2001). Most are epilithic or endolithic, and some are true endoliths with the capacity to dig and grow into calcium carbonates or sequestrate Ca-carbonates in their baeocytes (Garcia-Pichel et al., 2010; Benzerara et al., 2014; Guida and Garcia-Pichel, 2016) leading to marine and terrestrial carbonate erosion and deleterious effects on coral reef and bivalve ecology. *Hyella* is a euendolithic baeocytous genus characterized by cells surrounded by a firm sheath, and the thalli often form branching pseudofilaments that can grow on calcium carbonate substrates (Al-Thukair and Golubic, 1991; Al-Thukair, 2011; Brito et al., 2017). To date, thirteen genomes representing five baeocyte-forming genera have been reported (NCBI and IMG/JGI databases) but none from *Hyella* is available so far.

Beyond evolution and classification of the organisms, the growing interest in genomics of cyanobacteria is driven by the discovery of new drugs (Kleigrewe et al., 2015; Moss et al., 2016). A genome-mining study revealed that up to 70% of cyanobacterial genomes contain polyketide synthase (PKS) and non-ribosomal peptide synthetase (NRPS) pathways or hybrids of these two (Calteau et al., 2014). Molecules derived from these pathways constitute the majority of known cyanobacterial NPs, but only 20% of the gene clusters of the PKS and NRPS pathways could be assigned to known compounds (Calteau et al., 2014), highlighting a large number of orphan clusters, with products yet to be discovered. In addition, gene clusters involved in the ribosome-dependent synthesis and post-translationally modified peptides (RiPPs) are also present throughout the phylum (Shih et al., 2013). RiPPs, PKS and NRPS gene clusters are well represented within the few available baeocyte-forming cyanobacterial genomes, but little is known about these biosynthetic pathways and their products compared to any other cyanobacterial lineages investigated (Dittmann et al., 2015).

Previously, we isolated and characterized a new marine *Hyella* strain – *H. patelloides* LEGE 07179 – from a rocky beach on the North of Portugal (Brito et al., 2012, 2017), and a preliminary metabolomic analysis revealed the potential of this cyanobacterium to produce compounds related to neopeptin and antanapeptin as well as novel ones (Brito et al., 2015). The present study aimed at enlarging the baeocytous cyanobacterial genomic representation by sequencing for the first time the genome of *Hyella*. Moreover, a comprehensive comparative study with the available genomes of its closest relatives was performed to evaluate the distinctive characteristics of the genus. In addition, extensive analyses regarding NPs biosynthetic gene clusters were carried out to obtain an overview of their diversity and putative products.

## Results

**FIGURE 1:**
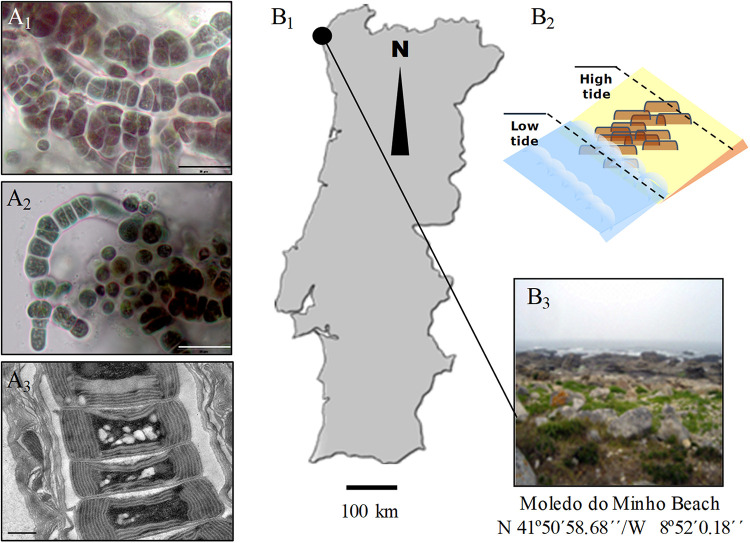
Light **(A_1_,A_2_)** and transmission electron **(A_3_)** micrographs of *Hyella patelloides* LEGE 07179. Geographical location **(B_1_)**, graphical representation **(B_2_)** and image of the sampling site **(B_3_)**. Scale bars: **(A_1_,A_2_)**, 20 μm; **(A_3_)**, 0.5 μm.

*Hyella patelloides* LEGE 07179 (hereafter referred to *H. patelloides*) (Figures 1A_1_–A_3_) was isolated from a *Patella* sp. shell collected from the intertidal zone of a rocky beach in the North of Portugal (Figures 1B_1_–B_3_). This species displays club-shaped or cylindrical cells that divide by multiple binary fission originating baeocytes, and cells/colonies are surrounded by a multi-layered sheath from which pseudofilaments can emerge, especially when growing in solid medium (Figures 1A_1_–A_3_).

### Genome Properties, Phylogeny and Comparative Analysis

The draft genome of *H. patelloides* has an estimated size of about 8.1 Mb assembled in 675 contigs, with a 37.57% GC content and a coverage of 107.8 times. The average contig size is 11946.2 bp and the N50 is 20427. Gene annotation revealed 8104 CDS with three rRNA genes and 50 tRNA genes. Despite using both 200 and 600-base-pair-read libraries, the genome, with a high proportion of repetitive sequences, could not be assembled into a single scaffold. In addition, *H*. *patelloides* genome was evaluated for its completeness and contamination level using CheckM (Parks et al., 2015). The results obtained (completeness – 99.2% and contamination level – 1.9%) highlight its quality (according to Parks et al., 2015).

The main characteristics of *H*. *patelloides* genome were compared to all baeocyte-forming cyanobacterial genomes available (Supplementary Table S1). The genome sizes of these strains range from 4.9 to 8.1 Mb with a low GC content (between 35 and 45%), despite their various origins (marine, freshwater, soil and hot and mineral springs). The majority of these genomes (obtained from axenic or non-axenic strains) remain in draft form, notably the largest ones (>10 scaffolds) (Supplementary Table S1).

**FIGURE 2:**
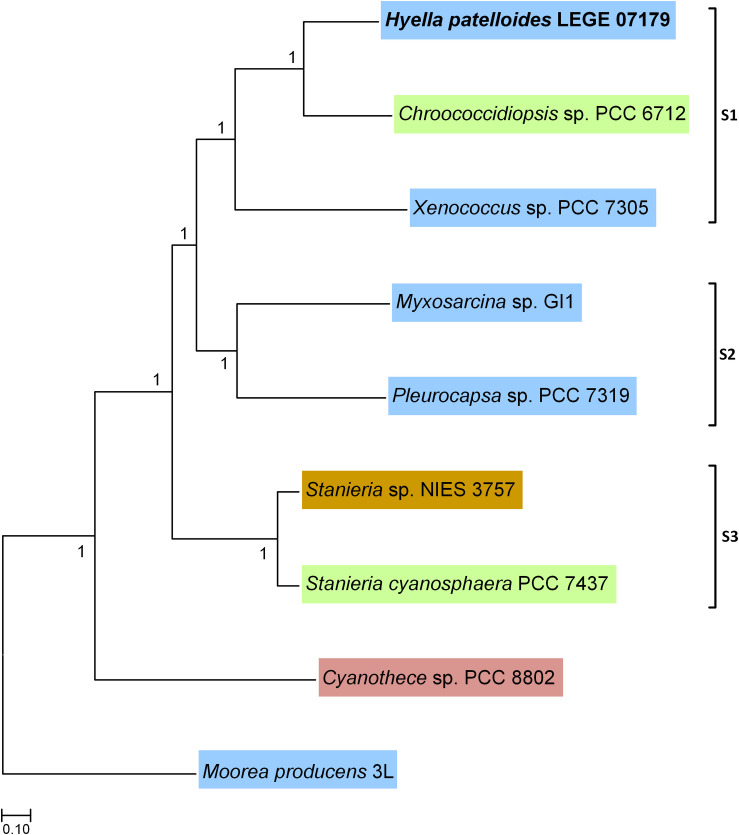
Bayesian phylogenetic tree based on 1209 concatenated orthologous genes shared between *Hyella patelloides* LEGE 07179 and the cyanobacterial strains selected for this analysis. *Moorea producens* 3L and *Cyanothece* sp. PCC 8802 were used to root the tree. Numbers along branches indicate posterior credibility probability values. The strains’ habitat is highlighted by different colors: Blue – marine; Green – freshwater; Brown – soil; Pink – rice field.

Based on the previous 16S rRNA gene phylogenetic study, *H. patelloides* is placed within the major baeocystous clade (Brito et al., 2017). Thus, we selected the following *H*. *patelloides* closest strains for the comparative and phylogenomic analysis: *Chroococcidiopsis* sp. PCC 6712, *Xenococcus* sp. PCC 7305, *Myxosarcina* sp. GI1, *Pleurocapsa* sp. PCC 7319, *Stanieria cyanosphaera* PCC 7437 and *Stanieria* sp. NIES 3757 as well as *Cyanothece* sp. PCC 8802 and *Moorea producens* 3L as outgroups. The phylogenomic analysis was based on 1209 orthologous genes (Supplementary Table S2 and Supplementary File S1), shared between the selected strains, and three sub-clusters appear clearly defined in the Bayesian phylogenetic tree: one composed by *H*. *patelloides*, *Chroococcidiopsis* sp. PCC 6712 and *Xenococcus* sp. PCC 7305 (S1), another by *Myxosarcina* sp. GI1 and *Pleurocapsa* sp. PCC 7319 (S2), and a third one including the two *Stanieria* strains (NIES 3757 and PCC 7437) (S3) (Figure 2). These sub-clusters are highly congruent with the previous 16S rRNA phylogeny (Brito et al., 2017), and are in line with other phylogenetic studies based on the concatenation of conserved proteins (Shih et al., 2013; Calteau et al., 2014). The two *Stanieria* are the most distantly related to *H*. *patelloides*, whereas the most closely related cyanobacterium is the freshwater *Chroococcidiopsis* sp. PCC 6712, however this strain is still highly divergent from *Hyella*. Indeed, using the same alignment of these 1209 orthologous genes, the synonymous changes per synonymous position (*Ks*) and non-synonymous changes per non-synonymous position (*Ka*) values between these two strains were estimated as 0.9043 (253971.3 synonymous positions analyzed) and 0.0888 (842237.6 non-synonymous positions analyzed), respectively. These values are between those obtained when comparing the two *Stanieria* species [*Ks* = 0.2828 (255486.8 synonymous positions analyzed) and *Ka* = 0.0259 (840722.1 non-synonymous positions analyzed)] and those obtained when comparing *Myxosarcina* sp. G1 and *Pleurocapsa* sp. PCC 7319 [(*Ks* = 1.8362 (258426.0 synonymous positions analyzed) and *Ka* = 0.1575 (837783.0 non-synonymous positions analyzed)] (Supplementary Table S3). Nevertheless, it is difficult to translate such divergence values into time (million years) since according to the Tajima’s relative rate tests (compares evolutionary rates between species), the different lineages are clearly evolving at different rates. The pattern is more evident when analyzing third codon position than amino acid differences, thus significant changes in mutation rate are the most likely explanation for our observations (Supplementary Table S4). It should also be noted that for most comparisons involving third codon positions only, saturation could have an important impact on the inferences, especially if different species have different codon preferences. When performing the test using amino acid differences, significantly different rates are observed in four comparisons, three of them involving *H*. *patelloides* (Supplementary Table S4). The lineage leading to *H*. *patelloides* is accumulating amino acid differences faster than the lineage leading to *Stanieria*, but slower than the lineages leading to *Xenococcus* sp. PCC 7305, thus analysis of the *H*. *patelloides* biology and genome could reveal new insights into cyanobacterial biology and diversity.

### Homologous Genes

When looking to the orthologous and paralogous genes shared between *H*. *patelloides* and the selected cyanobacterial strains, between 3244 (40%) and 3880 (47.9%) of *H*. *patelloides* genes have orthologs in another strain (Table 1). If both orthologs and paralogs are considered, in between 5480 and 5998 *H*. *patelloides* genes are recognized in the other strains studied (Table 1). The percentage of *H*. *patelloides* duplicated genes (27%) is higher than the ones observed for any other baeocyte-genomes, notably the ones of the same clade, *Chroococcidiopsis* sp. PCC 6712 (838 genes; 16.2% of the *Chroococcidiopsis* genes) and *Xenococcus* sp. PCC 7305 (1090 genes; 20.1% of the *Xenococcus* genes). However, it is closer to the percentage observed for *Myxosarcina* sp. GI1 (1593 genes; 24.4% of the *Myxosarcina* genes) and *Pleurocapsa* sp. PCC 7319 (1640 genes; 24.3% of the *Pleurocapsa* genes) (Supplementary Table S5). This is compatible with an independent loss of duplicated genes in the *Chroococcidiopsis* and *Xenococcus* lineages, a gain of duplicated genes in the *H*. *patelloides* lineage, but also with other more complex scenarios, involving, for instance, horizontal gene transfer (HGT). Remarkably, *H*. *patelloides* shares more genes with the more distantly related marine strains of the S2 clade (*Pleurocapsa* sp. PCC 7319 and *Myxosarcina* sp. GI1) than with the more closely related marine strain of the clade S1 (*Xenococcus* sp. PCC 7305). This could indicate a significant gene loss in the *Xenococcus* lineage but it is also compatible with HGT. In addition, there is a high number of *H*. *patelloides* genes (2106–2624) with no similarity to the genes described for each of the strains studied (Table 1). However, if all strains are considered as if they were a single one, only 794 *H. patelloides* genes do not show similarity to the other baeocyte-forming cyanobacterial strains studied, as if, by chance alone, *H. patelloides* retains genes that are lost in the other lineages.

**TABLE 1:** Number/percentage of orthologous and paralogous genes shared by *Hyella patelloides* LEGE 07179 and the selected baeocyte-forming cyanobacterial strains, as well as the number/percentage of *H*. *patelloides* genes with no similarity with the ones from those strains.

**Cyanobacterial strains**	**Orthologous**	**Paralogous**	**No similarity**
*Chroococcidiopsis* sp. PCC 6712	3619 (44.7%)	2256 (27.8%)	2229 (27.5%)
*Xenococcus* sp. PCC 7305	3320 (41.0%)	2200 (27.1%)	2584 (31.9%)
*Myxosarcina* sp. GI1	3573 (44.1%)	2291 (28.3%)	2240 (27.6%)
*Pleurocapsa* sp. PCC 7319	3880 (47.9%)	2118 (26.1%)	2106 (26.0%)
*Stanieria cyanosphaera* PCC 7437	3282 (40.5%)	2303 (28.4%)	2519 (31.1%)
*Stanieria* sp. NIES 3757	3244 (40.0%)	2236 (27.6%)	2624 (32.4%)

### Horizontal Gene Transfer

**FIGURE 3:**
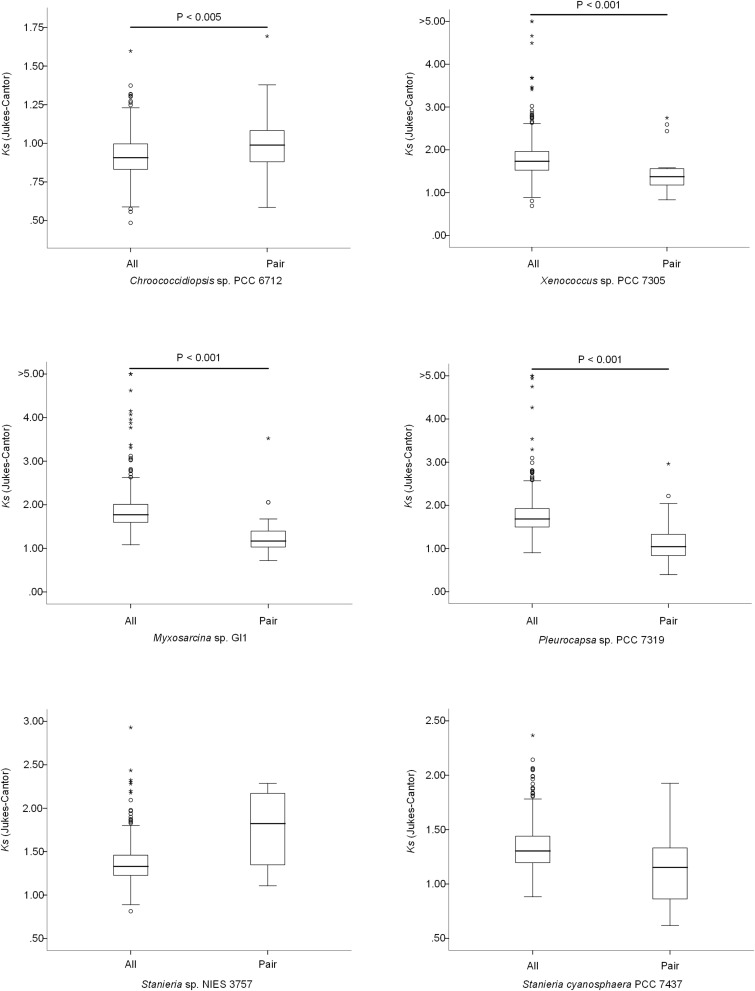
Comparison of the synonymous rate (Ks) distributions based on sets of 500 non-overlapping synonymous sites obtained using concatenated gene alignments. All – sets of genes for which a putative orthologous gene was found in all species; Pair – genes for which the ortholog was found only in *Hyella patelloides* LEGE 07179 and the analyzed strain.

**FIGURE 4:**
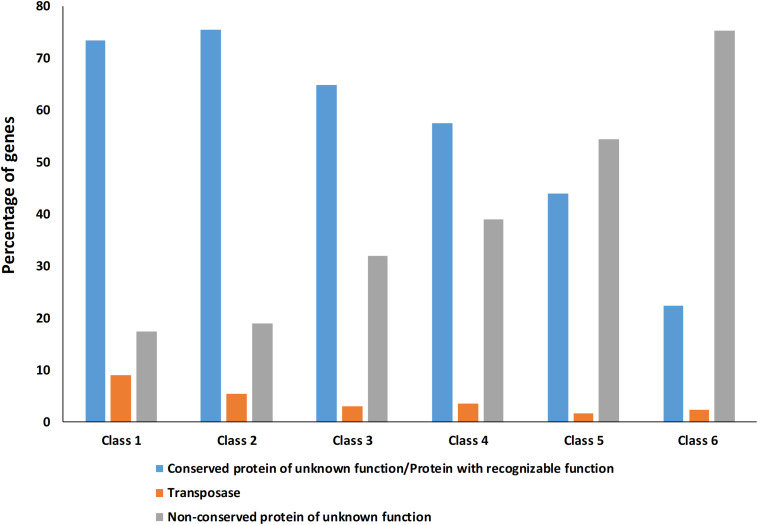
Percentage of *Hyella patelloides* LEGE 07179 genes identified as conserved protein of unknown function/protein with recognizable function, transposase or non-conserved protein of unknown function absent in one or more of the baeocyte-forming cyanobacteria studied. Class 1 – genes absent in one strain; Class 2 – genes absent in two strains; Class 3 – genes absent in three strains; Class 4 – genes absent in four strains; Class 5 – genes absent in five strains; Class 6 – genes absent in six strains (only present in *H*. *patelloides*).

**FIGURE 5:**
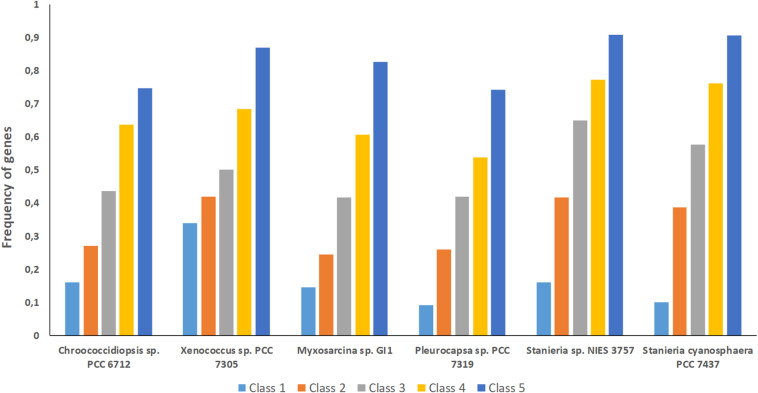
Frequency of genes from the different classes analyzed (1 to 5) per baeocyte-forming cyanobacterial strain studied. Class 1 – genes absent in one strain; Class 2 – genes absent in two strains; Class 3 – genes absent in three strains; Class 4 – genes absent in four strains; Class 5 – genes absent in five strains.

The high number of putative orthologs shared between *H*. *patelloides* and the other baeocyte-forming strains studied (Table 1) suggests horizontal gene transfer (HGT). To evaluate this transfer, the *Ks* values for a set of orthologs identified in all strains (“All”; Supplementary Table S2) were compared against the set of orthologs found in *H. patelloides* and another strain only (“Pair”; Supplementary Table S6). The dataset for “All” is the 1209 concatenated gene alignments with an average of 261835.7 synonymous sites, while the datasets for the *Ks* distributions under the label “Pair” for *Chroococcidiopsis* sp. PCC 6712, *Xenococcus* sp. PCC 7305, *Myxosarcina* sp. GI1, *Pleurocapsa* sp. PCC 7319, *Stanieria cyanosphaera* PCC 743, and *Stanieria* sp. NIES 3757 were based on 126, 62, 99, 185, 26, and 19 concatenated gene alignments, respectively (with 20274.8, 8366.7, 14767, 30910.2, 4798.08, and 1581.6, synonymous sites, respectively). If the pattern was created by HGT, the *Ks* value estimated for a “Pair” will be lower than the *Ks* estimated for “All.” In fact, this tendency is observed between *H*. *patelloides* and all marine strains (*Xenococcus* sp. PCC 7305, *Myxosarcina* sp. GI1 and *Pleurocapsa* sp. PCC 7319) and *Stanieria cyanosphaera* PCC 7437 (Figure 3), supporting the high probability of occurring HGT events between these strains. Then, we investigated if the putative orthologs shared between *H*. *patelloides* and the other baeocyte-forming strains have a recognizable function, since they could be “false gene predictions.” Therefore, the annotated genes were divided into different categories (conserved protein of unknown function/protein with recognizable function, transposase, and non-conserved protein of unknown function), and distributed into different classes taken into account their presence/absence in one or more of the strains studied [classes 1 to 6 correspond to genes that are absent in one (class 1), two (class 2), three (class 3), four (class 4), five (class 5), and six (class 6) strains]. Thus, it was possible to observe that a high percentage of these genes are annotated as conserved protein of unknown function or protein with recognizable function (notably genes that are only absent in one or two strains and so, present in the majority of them – Classes 1 and 2). However, when we look to the genes that are only present in three or less strains, this percentage slightly decreases (Classes 3 to 6) (Figure 4 and Supplementary Tables S7, S8). In addition, some genes are identified as “transposase” or “non-conserved protein of unknown function.” Taken into account the low percentage of “transposase” genes identified, the high number of putative orthologs found is not an artifact created by annotating mobile elements as *H*. *patelloides* genes (Figure 4 and Supplementary Tables S7, S8). Concerning the genes absent in six strains and thus only present in *H*. *patelloides* (Class 6), the majority of them are annotated as non-conserved protein of unknown function, but still there is a reasonable percentage of strain-specific genes identified as conserved protein/protein with a recognizable function (Figure 4 and Supplementary Tables S7, S8). Closely related species are expected to have more genes in common than distantly related ones. Therefore, we looked at whether when a gene is only absent in one or few strains, that strain(s) is usually the same one or not. Therefore, as can be observed in Figure 5, when a gene is absent just in one strain (Class 1), in the majority of the cases *Xenococcus* sp. PCC 7305 is that strain (light blue bar, Figure 5 and Supplementary Table S8). In addition, *Stanieria* strains and *Xenococcus* sp. PCC 7305 are the ones for which more genes are absent in the remaining classes (Classes 2 to 5). Although, this was expected for the *Stanieria* strains, since they are the ones more distantly related to *H*. *patelloides*, *Xenococcus* sp. PCC 7305 appeared as the strain with a higher number of “missing genes” within the group of baeocyte-forming strains studied. Interestingly, in the Class 5 (genes absent in five strains and thus, only present in *H*. *patelloides* and another strain), *Pleurocapsa* sp. PCC 7319 is the strain with the lowest frequency followed by the *H*. *patelloides* closest strain *Chroococcidiopsis* sp. PCC 6712 (Figure 5 and Supplementary Table S8). Thus, for the majority of the cases where *H. patelloides* genes are missing in other baeocyte-forming strains, they are still present in *Pleurocapsa* sp. PCC 7319.

**FIGURE 6:**
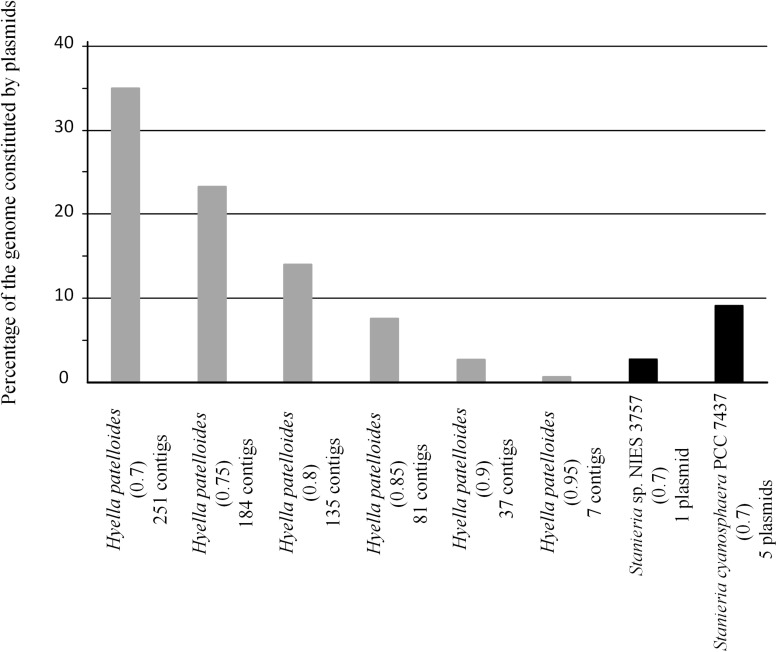
Fraction of *Hyella patelloides* LEGE 07179, *Stanieria cyanosphaera* PCC 7437 and *Stanieria* sp. NIES 3757 genomes constituted by plasmids, using PlasFlow with different cut-offs (number between brackets).

In addition, we addressed if the HGT events could be mainly related to plasmid transfer. Among the studied strains, only *Stanieria* spp. have their genomes assigned in chromosome and plasmids (*Stanieria cyanosphaera* PCC 7437: Chr – 5.04 Mb and 5 plasmids – 0.50 Mb (9.1%); *Stanieria* sp. NIES-3757: Chr – 5.32 Mb and 1 plasmid – 0.14 Mb (2.6%). Therefore, plasmid sequences in the *H*. *patelloides* genome, as well as in the other strains studied, were predicted using PlasFlow (default parameters, threshold = 0.7) (Supplementary Table S9). For both *Stanieria* strains, the fraction (base pairs) predicted to be located in plasmids is in line with the values reported in the databases. However, this is only true if the category “unclassified” is assumed to be “chromosome.” Therefore, for the remaining genomes, the same assumption was followed. Concerning to *H*. *patelloides*, 35% of its genome is predicted to be constituted by plasmids. This value is rather high, when compared to those from the other strains (0.4 to 21%), namely compared to *Stanieria* (less than 10%). Thus, in order to have a value for *H*. *patelloides*, in the same range of that reported for *Stanieria*, a threshold as high as 0.85 would have to be used (Figure 6). Genes for which an ortholog has been identified in all strains are often located in sequences predicted to be of plasmid origin, even when a high PlasFlow threshold value is used (Supplementary Table S10). This is an odd observation, since the same plasmid is unlikely to be present in all species, and the use of the traditional reverse BLAST for ortholog identification should not produce so many erroneous orthologous identifications. In fact, none of the 1209 genes from *Stanieria* sp. NIES-3757 that are present in the other strains, and were used in the phylogenetic analyses, are located in the single plasmid reported for this strain. Moreover, only two out of the 1209 genes from *Stanieria cyanosphaera* PCC 7437 are located in plasmid sequences [one from plasmid 1 (2503797950) and another from plasmid 3 (2503797623)] (Supplementary Table S2). Hence, the traditional reverse BLAST for ortholog identification seems to work well since, as expected, less than 0.17% of the genes present in all strains are located in *Stanieria* plasmids.

Furthermore, we looked into the genes present in *H*. *patelloides* and another strain only, and according to PlasFlow some of these genes (29 to 53%) are predicted to be located in plasmids, but the majority are in the “unclassified” category (Table 2). When considering the set of 19 genes predicted to be present in *H. patelloides* and *Stanieria* sp. NIES 3757, two genes are located in plasmid 1 (gene 879 predicted to be a pseudogene and WP_096388421.1_4869). When considering the 26 genes predicted to be present in *H. patelloides* and *Stanieria cyanosphaera* PCC 7437, nine genes are from plasmids (2503802167, 2503797825, 2503798028, 2503797810, 2503797825, and 2503798007 on plasmid 1; 2503797602, 2503797607, and 2503797643 on plasmid 3) (Supplementary Table S6). Therefore, as expected, since plasmids are unlikely to be present in all species, genes that are found in *H. patelloides* and another strain only, seem to have a much higher chance of being located in plasmids than the ones present in all species (*P* < 0.0005 and *P* < 0.000001 for *Stanieria* sp. NIES 3757 and *Stanieria cyanosphaera* PCC 7437, respectively; Fisher exact test). However, most sequences identified as of “plasmid origin” by PlasFlow show genes that are present in all species, as well as in *H. patelloides* and in a single strain (Supplementary Table S10). Moreover, *H. patelloides* genes that have orthologs in *Stanieria* plasmids can be located in scaffolds that also contain genes identified in all species. Therefore, it is not easy to be sure that the sequences identified as of plasmid origin by PlasFlow are correctly identified. However, these findings should be regarded as indicative rather than definitive, and support the inference that *H*. *patelloides* genome is constituted by chromosomes and plasmids and some of the genes present in this strain and another one only (from the set studied), are located in plasmids.

**TABLE 2:** Number of genes present in *Hyella patelloides* LEGE 07179 and in another baeocyte-forming cyanobacterial strain only, and their location in plasmid or chromosomal according to the PlasFlow prediction.

**Cyanobacterial strains**	**Chromosome**	**Plasmid**	**Unclassified**	**Total**
*Chroococcidiopsis* sp. PCC 6712	5	37	84	126
*Xenococcus* sp. PCC 7305	2	21	39	62
*Myxosarcina* sp. GI1	4	33	62	99
*Pleurocapsa* sp. PCC 7319	5	94	86	185
*Stanieria* sp. NIES-3757	1	10	8	19
*Stanieria cyanosphaera* PCC 7437	1	9	16	26

### COG Categories Distribution

The distribution of genes among the Clusters of Orthologous Groups (COG) functional categories, allowed to assign 58.9% of the *H*. *patelloides* genes (Supplementary Table S11), slightly less than those observed for the other baeocyte-forming cyanobacterial strains analyzed (between 64 and 68%). Interestingly, the seven genomes exhibit similarities in the COG functional category distribution. Thus, the percentage of genes assigned to the different functional categories is similar for all strains analyzed, independently of their genome size.

### Natural Product Biosynthetic Gene Clusters and Hydrocarbons Production

**FIGURE 7:**
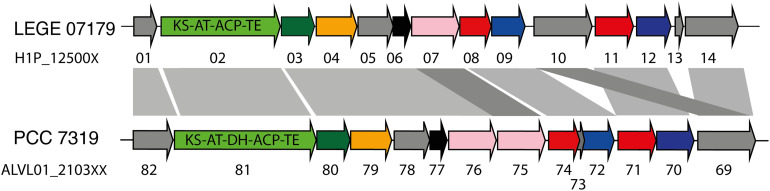
PKS Gene cluster shared by *Hyella patelloides* LEGE 07179 and *Pleurocapsa* sp. PCC 7319. The genes are color coded according to their putative function: green for PKS (with the domains listed), dark green for 3-oxoacyl synthase, orange for cytochrome P450, pink for NAD(P)-binding domain-containing protein, blue for sulfotransferase, red for transporter, black for methyltransferase and gray for unknown proteins. KS, ketosynthase; AT, acyltransferase; ACP, acyl carrier protein; TE, thioesterase; DH, dehydratase. Further details provided in Supplementary Table S13.

**FIGURE 8:**
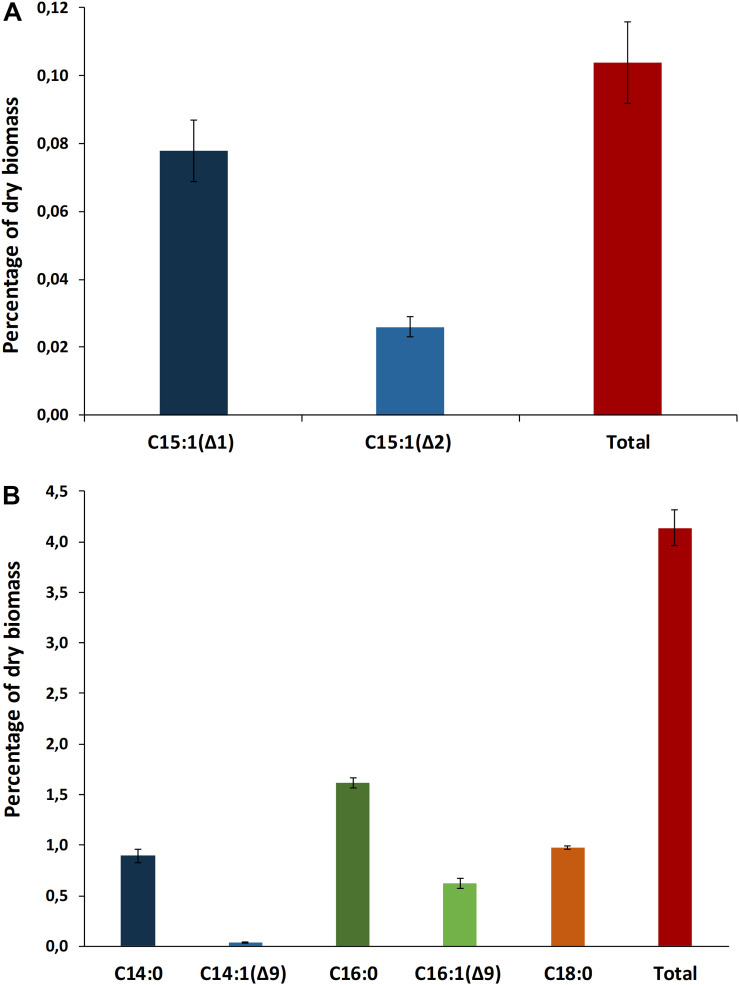
Hydrocarbon profile of *Hyella patelloides* LEGE 07179. Terminal olefins with chain length of C_15_ were identified **(A)**, and fatty acid composition of *Hyella patelloides* with chain lengths of C_14_, C_16_, and C_18_ were identified **(B)**. Number after the colon indicates the number of double bonds, while the number after the triangle indicates the position of the double bound. Data are the means of three biological replicates, and error bars represent standard deviations.

A total of 21 biosynthetic gene clusters (BGCs) involved in the production of natural products were identified in the *H*. *patelloides* genome. Interestingly, *H*. *patelloides* and *Pleurocapsa* sp. PCC 7319, both isolated from a marine organism shell (intertidal zone), harbor the highest number of predicted BGCs (21 and 23, respectively) (Supplementary Table S1). The *H*. *patelloides* BGCs include three polyketide synthase (PKS), four non-ribosomal peptide synthetase (NRPS), five hybrid PKS/NRPS, six RiPPs, and three terpenes. Remarkably, most of these BGCs displayed low similarity with the ones available in the antiSMASH database (Supplementary Table S12). However, two of *H*. *patelloides* BGCs have similarities with the ones identified in other cyanobacterial strains. The first one (PKS gene cluster) shares 13 genes in synteny with an orphan cluster identified in *Pleurocapsa* sp. PCC 7319. This shared cluster is constituted by one module of PKS (with a dehydratase domain in PCC 7319) and eleven diversified tailoring enzymes with putative function of methyl- and sulfotransferases and transporters (Figure 7 and Supplementary Table S13). Considering the high similarity of these two clusters (59%) and the close relationship between these strains, the characterization of the putative compound can be undertaken using both strains. The second BGC is a PKS gene cluster involved in the production of hydrocarbons, a terminal olefin synthase pathway (OLS pathway), which is also present in the other baeocyte-forming strains studied (Zhu et al., 2018). Afterward, the hydrocarbon composition of *H. patelloides* was further investigated, revealing that this cyanobacterial strain produces hydrocarbons with C_15_ chain length, such as 1-pentadecene (C_15:1_, Δ1) and 2-pentadecene (C_15:1_, Δ2) (Figure 8A). Furthermore, the investigation of the fatty acid substrates for the OLS pathway showed that this strain synthesizes C_14_, C_16_, and C_18_ fatty acids, being C_16_ the most abundant. In total, the amount of fatty acid in *H*. *patelloides* exceeds 4% of the Dry Cell Weight (DCW) (Figure 8B).

As stated above, the majority of the *H*. *patelloides* BGCs displayed low similarity with the databases. Thus, in order to evaluate if they could be strain-specific BGCs we run them through the BiG-SCAPE software (Navarro-Muñoz et al., 2020). In this analysis, the 104 predicted BGCs from *H*. *patelloides* and the baeocyte-forming strains (Supplementary Table S1), as well as the ones from the Minimum Information about a Biosynthetic Gene cluster (MIBiG) repository (Kautsar et al., 2020), were compared. Using 0.3 and 0.5 similarity cut-offs no connections between *H*. *patelloides* BGCs and the other strains were obtained. However, the analysis with a 0.7 cut-off revealed four BGCs (2 PKS, 1 RiPP and 1 terpene) shared between *H*. *patelloides* and other strains (Supplementary Table S14). Of those clusters, the 2 PKS correspond to the ones mentioned above: the one that shares 13 genes in synteny with the orphan *Pleurocapsa* sp. PCC 7319 cluster (Supplementary Figure S1); the other PKS is the BGC involved in the production of a hydrocarbon (OLS pathway). This gene cluster family (GCF) comprises BGCs from *Chroococcidiopsis* sp. PCC 6712, *Xenococcus* sp. PCC 7305 and *Myxosarcina* sp. GI and it is connected to another one composed by BGCs from both *Stanierias.* According to the GCF phylogenetic tree, the *H*. *patelloides* BGC is closely related to the ones from *Chroococcidiopsis* sp. PCC 6712 and *Xenococcus* sp. PCC 7305 (Supplementary Figure S2). Although *Pleurocapsa* sp. PCC harbors this BGC, it appeared as a single node. The other two BGCs includes a bacteriocin/lanthipeptide gene cluster (RiPP) shared with *Pleurocapsa* sp. PCC 7319 and a terpene shared with *Chroococcidiopsis* sp. PCC 6712 (Supplementary Figure S1). These BGCs do not display similarities to known compounds. In addition, two GCFs composed by BGCs from *H*. *patelloides* related with different known ones from MIBiG (namely cyanopeptolin, micropeptin, cyanopeptin, and hapalosin biosynthetic gene clusters) were also observed (Supplementary Table S14 and Supplementary Figure S2). The remaining *H*. *patelloides* BGCs appeared as unclustered individual nodes (singletons) representing each one a different family (Supplementary Table S14). Thus, *H*. *patelloides* displays 70% of strain-specific clusters that could be involved in the production of yet unknown compounds.

Additional genome mining analyses were performed in order to better characterize the *H*. *patelloides* BGCs. To target antibiotic producing BGCs, the cluster prioritization tool Antibiotic Resistance Target Seeker (ARTS) was used. ARTS identified 410 core/essential genes, 21 BGCs and 68 known resistance models. The core/essential genes identified with the ARTS criteria (duplication, BGC proximity, phylogeny and known resistance) are shown in Supplementary Table S15. Concerning the BGC proximity, this analysis revealed that 17 of the 21 BGCs harbored neighboring putative core (clusters 1, 4, 6, 7, 8, 9, 10, 14, 17, 18, and 19), known resistance (2, 13, 21) or both genes (clusters 5, 12, 15) (Supplementary Table S15 and Supplementary Figure S3), emphasizing the ones with higher number of hits. For instance, in cluster 12, besides the core and two known resistant genes (ABC_efflux and pentapeptide repeat family), one core known resistance gene (Gp_dh_C) was also identified. In cluster 7, three of the core genes identified are marked as duplicated and with incongruent phylogeny. Therefore, some of the *H*. *patelloides* BGCs are associated with resistance mechanisms and further studies are needed in order to identity putative antibiotic-producing BGCs.

## Discussion

Despite the increasing number of available cyanobacterial genomes, there is an unbalanced distribution of genome sequences within the phylum. In the current study, the first draft genome from a member of *Hyella* genus is presented, contributing to overcome the total lack of information regarding this genus and to increase the data on baeocyte-forming cyanobacteria. The genome binning approaches ensured the quality of the *H*. *patelloides* draft genome and the CheckM analysis also supported it. Taken into account the *H*. *patelloides* position within the phylogenetic tree depicted in the present study, this strain constitutes a new representative of the main baeocytous subclade (within B2) previously described (Shih et al., 2013). Its large genome has the highest number of predicted ORFs compared to any of the baeocyte-forming cyanobacteria (Supplementary Table S1). Consequently, it was conceivable that *H*. *patelloides* genome could have unique features due to the expansion of its gene repertoire. When looking to the distribution of the genes among COG functional categories, the percentage of *H*. *patelloides* genes assigned to the different COG functional categories was within those found for the other strains analyzed (Supplementary Table S11). Thus, despite its large genome, the different gene functional categories seem to have been equally expanded. Nevertheless, it was still conceivable that *H*. *patelloides* shows some unique features, not only due to the observed high gene number, but also because it is highly divergent from the most closely related strains (Figure 2). Furthermore, a large number of genes seem to have been horizontally transferred between *H. patelloides* and the baeocyte-forming strains, such as the marine *Pleurocapsa* sp. PCC 7319 (also isolated from a shell) and *Myxosarcina* sp. GI1 strains (Figures 3, 5). Actually, there are more common genes between *H*. *patelloides* and *Pleurocapsa* sp. PCC 7319 than with its closest relative *Chroococcidiopsis* sp. PCC 6712, a freshwater strain (Table 1 and Figure 5). Since, *H. patelloides, Pleurocapsa* sp. PCC 7319 and *Myxosarcina* sp. GI1 are all originating from a marine environment, the occurrence of horizontal gene transfer (HGT) events between these strains is plausible. Actually, plasmid sequences were predicted in the *H*. *patelloides* genome (Figure 6 and Supplementary Tables S9, S10) and, although one cannot be sure that these sequences are correctly assigned, some genes present in *H. patelloides* and in another strain only, are predicted to be located in plasmids (Table 2 and Supplementary Table S10). This might indicate that HGT events mainly involved plasmid transfer, between two distantly related species living in the same environment, supporting the hypothesis that *H. patelloides* has captured plasmids from distantly related species in the past. Still, *H*. *patelloides* possesses a considerable set of genes with no similarity to those present in the other six strains analyzed (794), but, although some of these genes are putative, there is a considerable percentage that are conserved or with a recognizable function (Figure 4 and Supplementary Tables S7, S8).

The genome-mining analysis reinforced the already suggested metabolic potential of *H*. *patelloides* (Brito et al., 2015). Remarkably, *H. patelloides* and *Pleurocapsa* sp. PCC 7319, both isolated from a marine organism shell, are the ones harboring the highest number of BGCs in their genomes (Supplementary Table S1). From the 21 identified in *H*. *patelloides*, more than half are PKS and NRPS clusters (3 PKS, 4 NRPS, and 5 PKS/NRPS hybrids). Since a high percentage correspond to strain-specific gene clusters, as observed by the BGCs similarity network analysis (Supplementary Table S14), we are probably facing new chemical scaffolds, corresponding to NPs with unique properties. In addition, according to ARTS prediction, some *H*. *patelloides* BGCs are colocalized with putative core/essential and/or resistance genes (Supplementary Table S15 and Supplementary Figure S3), highlighting putative antibiotic producing BGCs. Moreover, in some studies the detection of duplicated housekeeping genes colocalized with BGCs led to the discovery of antibiotic producing gene clusters (Tang et al., 2015).

The *H*. *patelloides* BGC displaying high similarity to the orphan cluster previously identified in *Pleurocapsa* sp. PCC 7319 (Figure 7) belongs to the CF-46 cluster family, only identified in five other cyanobacterial strains (Calteau et al., 2014). This cluster has similar gene content and organization with *Pleurocapsa* sp. PCC 7319 BGC, sharing 13 genes in synteny. This BGC is not linked to any known compound, as it happens for most of the baeocystous strain NPs. However, it is composed by one PKS followed by eleven tailoring genes, being more similar to the saxitoxin gene cluster than a long PKS gene cluster. Indeed, these genes might be working collectively to produce a family of compounds (as in saxitoxin and in some antibiotics) (Fischbach et al., 2009; Mihali et al., 2009). The gene clusters containing a single PKS, NRPS or hybrids thereof are common in cyanobacteria and, indeed, easily overlooked as considered as remnant gene clusters (Calteau et al., 2014). Therefore, it is highly interesting to investigate the common or related compounds produced by these two strains.

Furthermore, the BGC involved in the production of a terminal olefin, belongs to the CF-8 cluster family previously described (Calteau et al., 2014). The terminal olefin synthase (OLS pathway) is one of the two biosynthetic pathways that produce hydrocarbons from fatty acids identified in cyanobacteria (Mendez-Perez et al., 2011; Coates et al., 2014; Zhu et al., 2018). This pathway is composed by a large type I PKS with modular organization that includes the following domains organization: Fatty acyl-AMP ligase (FAAL), acyl carrier protein (ACP), ketosynthase (KS), acyltransferase (AT), ketoreductase (KR), ACP2, sulfotransferase (ST) and thioesterase (TE) (Mendez-Perez et al., 2011; Coates et al., 2014; Zhu et al., 2018). There is the indication that cyanobacterial strains harboring this pathway produced more hydrocarbons than those possessing the alternative one (Coates et al., 2014). Interestingly, the type of hydrocarbon produced by *H. patelloides* (C_15_ chain length, Figure 8A) was recently shown to be produced by *Chroococcidiopsis* sp. PCC 6712 and *Xenococcus* sp. PCC 7305 (Zhu et al., 2018). This data is in accordance with the phylogenetic analyses of the GCF, where the *H*. *patelloides* BGC is closely related with *Chroococcidiopsis* sp. PCC 6712 and *Xenococcus* sp. PCC 7305 BGCs. In addition, according to our phylogeny (Figure 2), these two strains are the closest relatives to *H*. *patelloides*. This data is in accordance with Zhu et al. (2018) that suggested a correlation between the hydrocarbon profile and phylogeny based on the OLS pathway. *H*. *patelloides* and the other two C_15_ terminal olefin-producing strains displayed the same fatty acid composition (Figure 8B). Moreover, the amount of C_14_ fatty acid produced by *H*. *patelloides* is higher and in line with the values observed for *Cyanobacterium stanieri* PCC 7202, *Geminocystis herdmanii* PCC 6308 and *Chroococcidiopsis* sp. PCC 6712 (Zhu et al., 2018). The amount of total fatty acid in *H*. *patelloides* (>4% of DCW) is also higher, when compared to other terminal olefin-producing cyanobacterial strains (∼3% of DCW) (Zhu et al., 2018).

In summary, the first genome sequence of a cyanobacterium belonging to the baeocyte-forming genus *Hyella*, and the comparative analysis with its closest cyanobacterial counterparts, revealed its distinctiveness and, in particular, its unique biosynthetic potential (with more than twenty natural products BGCs identified). We found orphan and strain-specific BGCs that could be involved in the production of novel NPs and others could be studied as potential drug targets. Therefore, additional studies are needed to further characterize these clusters, as well as to identify and characterize the compounds produced and match them with the corresponding BGCs. For the BGC with a predicted product (terminal olefin) it was possible to quantify and characterize the hydrocarbon produced. Since terminal olefins have promising applications as advanced biofuels this molecular mechanism can be explored, for instance, using cell factories and a synthetic biology approach to increase the yield and become cost competitive.

## Materials and Methods

### Organism and Culture Conditions

*Hyella patelloides* LEGE 07179 was previously isolated from a *Patella* sp. shell collected in the intertidal zone of the rocky beach in the North of Portugal (Brito et al., 2012, 2017), and the unicyanobacterial culture is deposited at LEGE Culture Collection (LEGE CC) located at CIIMAR, Matosinhos, Portugal (Ramos et al., 2018). This strain is maintained in MN medium (Rippka, 1988) supplemented with 10 μg mL^–1^ of vitamin B_12_ and kept at 25°C, under a 16 h light (15–25 μmol photons m^–2^ s^–1^)/8 h dark regimen.

### Genome Sequencing and Assembly

Genomic DNA was extracted using the phenol/chloroform method described previously (Tamagnini et al., 1997), with the exception that the first aqueous phase was run through a Phase Lock Gel^TM^ tube (5 Prime, Hilden, Germany) prior to chloroform extraction and DNA precipitation. Genomic DNA was sequenced at the Genomics Core Facility at the i3S institute (GENCORE), Porto. Libraries of 200 and 600-base-pair-reads were sequenced using an Ion Torrent S5^TM^ XL Sequencer (Thermo Fisher Scientific). As the unicyanobacterial culture of *Hyella* was not axenic, its genomic data set was treated as a metagenome, and further binned to obtain cyanobacterial-specific contigs. The reads were initially assembled using the SPAdes v3.11.1 (Bankevich et al., 2012) and the ion-torrent specific option, the built-in read correction tool, and K-mer sizes 21, 33, 55, and 77. Contigs shorter than 1 kb were discarded resulting in a final set of 4818 contigs. A local BLASTn search using the 16S rRNA gene sequence of an uncultured marine cyanobacterium (JX477009) was performed and evidence for a minimum of nine different organisms was obtained. Besides the 16S rRNA gene sequence for *H. patelloides*, eight different bacteria were identified. Subject sequences were retrieved and a BLASTn analysis against the nt database at NCBI was carried out, revealing that the contaminants were from the Proteobacteria and Cytophaga-Flavobacterium-Bacteroides (CFB) groups. Thus, a local BLASTx was performed against a database containing the predicted proteomes of cyanobacteria (340 proteomes), proteobacteria (41109 proteomes) and CFB (1599 proteomes), and the first 20 hits were retrieved. 595 of the contigs displayed all hits with cyanobacterial proteins and they were considered as “cyanobacterial contigs” and retrieved. In addition, some contigs exhibited mostly hits with cyanobacterial proteins, and they were manually curated. 80 of them displayed a coverage compatible with their putative origin (higher than 70) and an identity much higher with cyanobacterial proteins than with CFB/proteobacteria and for this reason they were also retrieved. The remaining ones were considered as contaminants and were discarded.

To validate the previous approach and assure the accuracy and quality of the draft genome, the pegi3s Docker image^1^ of the automated binning tool MaxBin 2.0 (Wu et al., 2016) was also used and the majority of the cyanobacterial contigs (98%) are congruent, supporting our results (Supplementary Figure S4). However, there were 11 contigs that MaxBin 2.0 considered as non-cyanobacterial, although they display higher Blast identity and coverage with cyanobacteria than with other bacteria (Supplementary Figure S5A). There were also 27 contigs that MaxBin 2.0 considered as cyanobacterial ones but that show a high Blast identity and coverage both with cyanobacterial and non-cyanobacterial organisms (Supplementary Figure S5B). Thus, and in order to not wrongly infer horizontal gene transfer events (see section “Results”) these 27 contigs were not included.

The *H*. *patelloides* genome was submitted to the MicroScope platform v3.13.4 (Vallenet et al., 2019) for automatic annotation, and to evaluate genome completion and contamination using CheckM (Parks et al., 2015). The PlasFlow v1.1 was also used to predict plasmid sequences (Krawczyk et al., 2018; a Docker image is available at see text footnote 1).

*Hyella patelloides* draft genome has been deposited to the European Nucleotide Archive (ENA) under the study accession number PRJEB28569.

### Homologous Genes and Phylogenomic Analysis

Based on the previous 16S rRNA gene phylogenetic study, *H. patelloides* is placed within the major baeocystous clade (Brito et al., 2017). Thus, *H*. *patelloides* genome was compared with each one of the following strains: *Chroococcidiopsis* sp. PCC 6712, *Xenococcus* sp. PCC 7305, *Myxosarcina* sp. GI1, *Pleurocapsa* sp. PCC 7319, *Stanieria cyanosphaera* PCC 7437 and *Stanieria* sp. NIES 3757 as well as *Cyanothece* sp. PCC 8802 and *Moorea producens* 3L as outgroups.

Orthologous and paralogous genes were identified using the traditional reverse BLAST, also known as reciprocal best hits (Moreno-Hagelsieb and Latimer, 2008). Briefly, CDSs from *H*. *patelloides* were used as query and CDSs from each one of the selected strains as subject in a local tblastx search (expect value of 0.05). The first CDS hit from each strain was retrieved and used as query and the CDSs of *H*. *patelloides* as subject, and again the first hit was retrieved. If the first tblastx search produces no hits, the query CDS from *H*. *patelloides* was labeled as “no similarity.” If the second tblastx search returns a sequence that is different from the original query sequence then the original query was labeled as “paralog”; if it returns the same sequence as the original one, it was labeled as “ortholog.”

Out of the 1670 orthologous genes identified, 1209 produced multiple of three alignments (Supplementary Table S2; see Supplementary File S1 for the corresponding Fasta file showing the alignment of the concatenated sequences). Nucleotide alignments that were not multiple of three indicate that at least one sequence has a frameshift and thus those alignments were not used. For these analyses, Clustal Omega (Sievers et al., 2011) was used as the alignment algorithm and SEDA^2^ was used to select the multiple of three alignments. Therefore, the phylogenomic analysis was performed using the 1209 orthologous concatenated genes present in the selected strains and a Bayesian phylogenetic tree was obtained using MrBayes (Ronquist et al., 2012). The model of sequence evolution used was the General Time Reversible (GTR), allowing for among-site rate variation and a proportion of invariable sites. This was the selected model when using the Akaike information criterion (AIC), as implemented in jModelTest 2 (Darriba et al., 2012; a Docker image is available at see text footnote 1). Third codon positions were allowed to have a gamma distribution shape parameter different from that of first and second codon positions. Two independent runs of 1 000 000 Markov chain Monte Carlo generations with four chains each (one cold and three heated chains) were set up. The average standard deviation of split frequencies was below 0.000001. Moreover, the potential scale reduction factor for every parameter was about 1.00 showing that convergence has been achieved. Trees were sampled every 100th generation and the first 2500 samples were discarded (burn-in). The remaining trees were used to compute the Bayesian posterior probabilities of each clade of the consensus tree.

Genes identified in *H*. *patelloides* and another strain only, are listed in Supplementary Table S6. The corresponding Fasta file showing the alignment of the concatenated sequences can be found in Supplementary Files S2–S7. The files have been used for the divergence analyses.

The number of non-synonymous changes per non-synonymous position (*Ka*) and the number of synonymous changes per synonymous position (*Ks*) values have been estimated using the DnaSP software (Rozas et al., 2017). The non-parametric Mann–Whitney test was used in order to determine whether the two samples could have been obtained from the same *Ks* distribution. Tajima’s relative rate tests were performed using the Mega 7 software (Kumar et al., 2016).

### COG Categories Distribution

For each baeocyte-forming cyanobacterial strain, we examined how protein-coding genes are distributed in the various functional categories. Therefore, their distribution within the Clusters of Orthologous Groups (COG) was performed using the COGnitor tool (Tatusov et al., 1997), available at MicroScope platform (Vallenet et al., 2019).

### Natural Product Biosynthetic Gene Clusters Analysis

Natural product biosynthetic gene clusters (BGCs) of *H*. *patelloides* were identified using the genome mining software antiSMASH v5.0 (Blin et al., 2019). Subsequently, the identified BGCs were analyzed and compared against available cyanobacterial genomes using the MicroScope platform (Vallenet et al., 2019).

BiG-SCAPE (Biosynthetic Gene Similarity Clustering and Prospecting Engine) software v1.0.0^3^ (Navarro-Muñoz et al., 2020) was used to perform the biosynthetic gene cluster networking analyses. The dataset (input) included the 104 predicted BGCs (using antiSMASH v5.0) from *H*. *patelloides* and the baeocyte-forming strains studied here. The MIBiG parameter was set to include the MIBiG repository v1.4 (Kautsar et al., 2020). Analyses with different cut-offs of 0.3, 0.5, and 0.7 were performed. Phylogenetic trees inferring the evolutionary relationships of BGCs within each gene cluster family (GCF) provided by CORASON were generated within the BiG-SCAPE analysis.

The Antibiotic Resistance Target Seeker (ARTS) 2.0 tool (Alanjary et al., 2017) was used to target putative antibiotic producing BGCs (screening of putative resistance genes), using the default mode.

### Hydrocarbon Extraction

Hydrocarbons were extracted as previously reported (Tan et al., 2011; Zhu et al., 2018) with some modifications. 30 mg of *H. patelloides* lyophilized biomass were resuspended in 10 mL of sterile deionized water, homogenized using a 20 mL tissue homogenizer, and lysed by sonication. The lysate was extracted using 10 mL of chloroform-methanol (v/v, 2:1) for 2 h at room temperature. Prior to extraction, 30 μg of eicosane (C_20:0_) was added to the cell lysate as an internal standard. The organic phase was separated by centrifugation (8 000 × *g*, 5 min), and the extract was dried under a nitrogen stream at 55°C. The residue containing the hydrocarbons was redissolved in 1 mL of n-hexane and analyzed as previously reported (Zhu et al., 2018).

### Total Lipid Extraction and Methyl Esterification of Fatty Acids

Total lipids were extracted as previously reported (Lang et al., 2011; Tan et al., 2011; Zhu et al., 2018) with some modifications. 20 mg of *H. patelloides* lyophilized biomass were resuspended in 2 mL of sterile deionized water and homogenized using a 5 mL tissue homogenizer. Prior to extraction, 50 μg of nonadecanoic acid (C_19:0_) was added to the cell suspension as the internal standard. The samples were extracted using 4 mL of chloroform/methanol (v/v, 1:1), followed by homogenization using a vortex. The lower organic phase was separated by centrifugation (10 000 × *g*, 5 min), transferred into a 15 mL esterification tube, and dried under a nitrogen stream at 55°C. Then, 2 mL of 0.4 M KOH-methanol solution was added, and the mixture was incubated at 60°C for 1 h, allowing transesterification of lipid-bound fatty acids to the corresponding fatty acid methyl esters (FAMEs). Afterward, 4 mL of HCl/CH_3_OH (v/v, 1:9) were added to the mixture, and incubated at 60°C for 20 min. Finally, 2 mL of n-hexane and 3 mL of 5 M NaCl were added and gently mixed, and after keeping the mixture at room temperature for 20 min, the FAMEs (upper hexane phases) were transferred to sample vials and analyzed as previously reported (Zhu et al., 2018).

## Data Availability Statement

The datasets generated for this study can be found in the European Nucleotide Archive (ENA) under the study accession number PRJEB28569.

## Author Contributions

ÂB performed the experiments. ÂB, JV, CV, MG, PT, PL, and VR analyzed and interpreted the data. ÂB, JV, and CV assembled the genome and carried out the phylogenetic and genomic comparative analysis. TZ performed the hydrocarbon analysis. PT, MG, XL, and VV conceived and designed the study. All authors discussed, revised, and approved the final manuscript.

## Conflict of Interest

The authors declare that the research was conducted in the absence of any commercial or financial relationships that could be construed as a potential conflict of interest.

## Footnotes

1 https://pegi3s.github.io/dockerfiles/

2 https://www.sing-group.org/seda/

3 https://git.wageningenur.nl/medema-group/BiG-SCAPE

## References

[B1] Alanjary M. Kronmiller B. Adamek M. Blin K. Weber T. Huson D. (2017). The Antibiotic Resistant Target Seeker (ARTS), an exploration engine for antibiotic cluster prioritization and novel drug target discovery. *Nucleic Acids Res*. 45 W42–W48. 28472505 10.1093/nar/gkx360 PMC5570205

[B2] Al-Thukair A. A. (2011). Calculating boring rate of endolithic cyanobacteria *Hyella immanis* under laboratory conditions. *Int. Biodeter. Biodegr.* 65 664–667. 10.1016/j.ibiod.2011.03.009

[B3] Al−Thukair A. A. Golubic S. (1991). New endolithic cyanobacteria from the arabian gulf. I. *Hyella immanis* SP. NOV. 1. *J. Phycol.* 27 766–780. 10.1111/j.0022-3646.1991.00766.x

[B4] Bankevich A. Nurk S. Antipov D. Gurevich A. A. Dvorkin M. Kulikov A. S. (2012). SPAdes: a new genome assembly algorithm and its applications to single-cell sequencing. *J. Comput. Biol.* 19 455–477. 10.1089/cmb.2012.0021 22506599 PMC3342519

[B5] Benzerara K. Skouri-Panet F. Li J. Férard C. Gugger M. Laurent T. (2014). Intracellular Ca-carbonate biomineralization is widespread in cyanobacteria. *P. Natl. Acad. Sci. U.S.A.* 111 10933–10938. 10.1073/pnas.1403510111 25009182 PMC4121779

[B6] Blin K. Shaw S. Steinke K. Villebro R. Ziemert N. Lee S. Y. (2019). antiSMASH 5.0: updates to the secondary metabolite genome mining pipeline. *Nucleic Acids Res*. 47 W81–W87. 31032519 10.1093/nar/gkz310 PMC6602434

[B7] Brito Â Gaifem J. Ramos V. Glukhov E. Dorrestein P. C. Gerwick W. H. (2015). Bioprospecting Portuguese Atlantic coast cyanobacteria for bioactive secondary metabolites reveals untapped chemodiversity. *Algal Res.* 9 218–226. 10.1016/j.algal.2015.03.016

[B8] Brito Â Ramos V. Mota R. Lima S. Santos A. Vieira J. (2017). Description of new genera and species of marine cyanobacteria from the portuguese atlantic coast. *Mol. Phylogenet. Evol.* 111 18–34. 10.1016/j.ympev.2017.03.006 28279808

[B9] Brito Â Ramos V. Seabra R. Santos A. Santos C. L. Lopo M. (2012). Culture-dependent characterization of cyanobacterial diversity in the intertidal zones of the Portuguese coast: a polyphasic study. *Syst. Appl. Microbiol.* 35 110–119. 10.1016/j.syapm.2011.07.003 22277323

[B10] Calteau A. Fewer D. P. Latifi A. Coursin T. Laurent T. Jokela J. (2014). Phylum-wide comparative genomics unravel the diversity of secondary metabolism in cyanobacteria. *BMC Genomics* 15:977. 10.1186/1471-2164-15-977 25404466 PMC4247773

[B11] Castenholz R. W. (2001). “Phylum BX. cyanobacteria. oxygenic photosynthetic bacteria,” in *Bergey’s Manual of Systematic Bacteriology*, 2nd Edn, eds Boone D. R. Castenholz R. W. Garrity G. (New York, NY: Springer), 473–599. 10.1007/978-0-387-21609-6_27

[B12] Coates R. C. Podell S. Korobeynikov A. Lapidus A. Pevzner P. Sherman D. H. (2014). Characterization of cyanobacterial hydrocarbon composition and distribution of biosynthetic pathways. *PLoS One* 9:e85140. 10.1371/journal.pone.0085140 24475038 PMC3903477

[B13] Darriba D. Taboada G. L. Doallo R. Posada D. (2012). jModelTest 2: more models, new heuristics and parallel computing. *Nat. Methods* 9:772. 10.1038/nmeth.2109 22847109 PMC4594756

[B14] Deruyter Y. S. Fromme P. (2008). “Molecular structure of the photosynthetic apparatus,” in *The Cyanobacteria: Molecular biology, genomics and evolution*, eds Herrero A. Flores E. (Norfolk: Caister Academic Press), 217–269.

[B15] Díez B. Nylander J. A. Ininbergs K. Dupont C. L. Allen A. E. Yooseph S. (2016). Metagenomic analysis of the Indian ocean picocyanobacterial community: structure, potential function and evolution. *PLoS One* 11:e0155757. 10.1371/journal.pone.0155757 27196065 PMC4890579

[B16] Dittmann E. Gugger M. Sivonen K. Fewer D. P. (2015). Natural product biosynthetic diversity and comparative genomics of the cyanobacteria. *Trends Microbiol.* 23 642–652. 10.1016/j.tim.2015.07.008 26433696

[B17] Fischbach M. A. Walsh C. T. Clardy J. (2009). The evolution of gene collectives: how natural selection drives chemical innovation. *Proc. Natl. Acad. Sci. U.S.A*. 106:1679 10.1073/pnas.0812594106 PMC2290807 18216259

[B18] Flombaum P. Gallegos J. L. Gordillo R. A. Rincón J. Zabala L. L. Jiao N. (2013). Present and future global distributions of the marine Cyanobacteria *Prochlorococcus* and *Synechococcus*. *Proc. Natl. Acad. Sci. U.S.A.* 110 9824–9829. 23703908 10.1073/pnas.1307701110 PMC3683724

[B19] Garcia-Pichel F. Belnap J. Neuer S. Schanz F. (2003). Estimates of global cyanobacterial biomass and its distribution. *Algol. Stud.* 109 213–227. 10.1127/1864-1318/2003/0109-0213

[B20] Garcia-Pichel F. Ramírez-Reinat E. Gao Q. (2010). Microbial excavation of solid carbonates powered by P-type ATPase-mediated transcellular Ca^2+^ transport. *Proc. Natl. Acad. Sci. U.S.A.* 107 21749–21754. 10.1073/pnas.1011884108 21115827 PMC3003062

[B21] Guida B. S. Garcia-Pichel F. (2016). Extreme cellular adaptations and cell differentiation required by a cyanobacterium for carbonate excavation. *Proc. Natl. Acad. Sci. U.S.A.* 113 5712–5717. 10.1073/pnas.1524687113 27140633 PMC4878501

[B22] Kautsar S. A. Blin K. Shaw S. Navarro-Muñoz J. C. Terlouw B. R. van der Hooft J. J. (2020). MIBiG 2.0: a repository for biosynthetic gene clusters of known function. *Nucleic Acids Res*. 48 D454–D458. 31612915 10.1093/nar/gkz882 PMC7145714

[B23] Kleigrewe K. Almaliti J. Tian I. Y. Kinnel R. B. Korobeynikov A. Monroe E. A. (2015). Combining mass spectrometric metabolic profiling with genomic analysis: a powerful approach for discovering natural products from cyanobacteria. *J. Nat. Prod.* 78 1671–1682. 10.1021/acs.jnatprod.5b00301 26149623 PMC4681511

[B24] Krawczyk P. S. Lipinski L. Dziembowski A. (2018). PlasFlow: predicting plasmid sequences in metagenomic data using genome signatures. *Nucleic Acids Res.* 46:e35. 10.1093/nar/gkx1321 29346586 PMC5887522

[B25] Kumar S. Stecher G. Tamura K. (2016). MEGA7: molecular evolutionary genetics analysis version 7.0 for bigger datasets. *Mol. Biol. Evol.* 33 1870–1874. 10.1093/molbev/msw054 27004904 PMC8210823

[B26] Lang I. Hodac L. Friedl T. Feussner I. (2011). Fatty acid profiles and their distribution patterns in microalgae: a comprehensive analysis of more than 2000 strains from the SAG culture collection. *BMC Plant Biol.* 11:124. 10.1186/1471-2229-11-124 21896160 PMC3175173

[B27] Mendez-Perez D. Begemann M. B. Pfleger B. F. (2011). Modular synthase-encoding gene involved in α-olefin biosynthesis in *Synechococcus* sp. strain PCC 7002. *Appl. Environ. Microbiol.* 77 4264–4267. 10.1128/aem.00467-11 21531827 PMC3131656

[B28] Mihali T. K. Kellmann R. Neilan B. A. (2009). Characterisation of the paralytic shellfish toxin biosynthesis gene clusters in *Anabaena circinalis* AWQC131C and *Aphanizomenon sp*. NH-5. *BMC Biochem.* 10:8. 10.1186/1471-2091-10-8 19331657 PMC2679770

[B29] Moreno-Hagelsieb G. Latimer K. (2008). Choosing BLAST options for better detection of orthologs as reciprocal best hits. *Bioinformatics* 24 319–324. 10.1093/bioinformatics/btm585 18042555

[B30] Moss N. A. Bertin M. J. Kleigrewe K. Leão T. F. Gerwick L. Gerwick W. H. (2016). Integrating mass spectrometry and genomics for cyanobacterial metabolite discovery. *J. Ind. Microbiol. Biotechnol.* 43 313–324. 10.1007/s10295-015-1705-7 26578313 PMC5065021

[B31] Navarro-Muñoz J. C. Selem-Mojica N. Mullowney M. W. Kautsar S. Tryon J. H. Parkinson E. I. (2020). A computational framework to explore large-scale biosynthetic diversity. *Nat. Chem. Biol.* 16 60–68. 31768033 10.1038/s41589-019-0400-9 PMC6917865

[B32] Nunnery J. K. Mevers E. Gerwick W. H. (2010). Biologically active secondary metabolites from marine cyanobacteria. *Curr. Opin. Biotechnol.* 21 787–793. 10.1016/j.copbio.2010.09.019 21030245 PMC3034308

[B33] Parks D. H. Imelfort M. Skennerton C. T. Hugenholtz P. Tyson G. W. (2015). CheckM: assessing the quality of microbial genomes recovered from isolates, single cells, and metagenomes. *Genome Res.* 25 1043–1055. 10.1101/gr.186072.114 25977477 PMC4484387

[B34] Ramos V. Morais J. Castelo-Branco R. Pinheiro Â Martins J. Regueiras A. (2018). Cyanobacterial diversity held in microbial biological resource centers as a biotechnological asset: the case study of the newly established LEGE culture collection. *J. Appl. Phycol.* 30 1437–1451. 10.1007/s10811-017-1369-y 29899596 PMC5982461

[B35] Rippka R. (1988). “Isolation and purification of cyanobacteria,” in *Method. Enzymol*, eds Packer L. Glazer A. N. (San Diego, CA: Academic Press), 3–27. 10.1016/0076-6879(88)67004-2 3148836

[B36] Ronquist F. Teslenko M. Van Der Mark P. Ayres D. L. Darling A. Höhna S. (2012). MrBayes 3.2: efficient Bayesian phylogenetic inference and model choice across a large model space. *Syst. Biol.* 61 539–542. 10.1093/sysbio/sys029 22357727 PMC3329765

[B37] Rozas J. Ferrer-Mata A. Sánchez-Delbarrio J. C. Guirao-Rico S. Librado P. Ramos-Onsins S. E. (2017). DnaSP 6: DNA sequence polymorphism analysis of large data sets. *Mol. Biol. Evol.* 34 3299–3302. 10.1093/molbev/msx248 29029172

[B38] Shih P. M. Wu D. Latifi A. Axen S. D. Fewer D. P. Talla E. (2013). Improving the coverage of the cyanobacterial phylum using diversity-driven genome sequencing. *Proc. Natl. Acad. Sci. U.S.A.* 110 1053–1058. 10.1073/pnas.1217107110 23277585 PMC3549136

[B39] Sievers F. Wilm A. Dineen D. Gibson T. J. Karplus K. Li W. (2011). Fast, scalable generation of high−quality protein multiple sequence alignments using Clustal Omega. *Mol. Syst. Biol.* 7:539. 10.1038/msb.2011.75 21988835 PMC3261699

[B40] Sivonen K. Leikoski N. Fewer D. P. Jokela J. (2010). Cyanobactins–ribosomal cyclic peptides produced by cyanobacteria. *Appl. Microbiol. Biotechnol.* 86 1213–1225. 10.1007/s00253-010-2482-x 20195859 PMC2854353

[B41] Tamagnini P. Troshina O. Oxelfelt F. Salema R. Lindblad P. (1997). Hydrogenases in *Nostoc* sp. strain PCC 73102, a strain lacking a bidirectional enzyme. *Appl. Environ. Microbiol.* 63 1801–1807. 10.1128/aem.63.5.1801-1807.1997 16535596 PMC1389151

[B42] Tan X. Yao L. Gao Q. Wang W. Qi F. Lu X. (2011). Photosynthesis driven conversion of carbon dioxide to fatty alcohols and hydrocarbons in cyanobacteria. *Metab. Eng.* 13 169–176. 10.1016/j.ymben.2011.01.001 21220042

[B43] Tang X. Li J. Millán-Aguiñaga N. Zhang J. J. O’Neill E. C. Ugalde J. A. (2015). Identification of thiotetronic acid antibiotic biosynthetic pathways by target-directed genome mining. *ACS Chem. Biol.* 10 2841–2849. 10.1021/acschembio.5b00658 26458099 PMC4758359

[B44] Tatusov R. L. Koonin E. V. Lipman D. J. (1997). A genomic perspective on protein families. *Science* 278 631–637. 10.1126/science.278.5338.631 9381173

[B45] Vallenet D. Calteau A. Dubois M. Amours P. Bazin A. Beuvin M. (2019). MicroScope: an integrated platform for the annotation and exploration of microbial gene functions through genomic, pangenomic and metabolic comparative analysis. *Nucleic Acids Res.* 48 D579–D589. 10.1093/nar/gkz926 PMC7145621 31647104

[B46] Wu Y.-W. Simmons B. A. Singer S. W. (2016). MaxBin 2.0: an automated binning algorithm to recover genomes from multiple metagenomic datasets. *Bioinformatics* 32 605–607. 10.1093/bioinformatics/btv638 26515820

[B47] Zehr J. P. (2011). Nitrogen fixation by marine cyanobacteria. *Trends Microbiol.* 19 162–173. 10.1016/j.tim.2010.12.004 21227699

[B48] Zhu T. Scalvenzi T. Sassoon N. Lu X. Gugger M. (2018). Terminal olefin profiles and phylogenetic analyses of olefin synthases in diversified cyanobacterial species. *Appl. Environ. Microbiol.* 84 e425–e418. 10.1128/AEM.00425-18 PMC6007117 29728380

