# Supplementary material for: Comparative Genomics Discloses the Uniqueness and the Biosynthetic Potential of the Marine Cyanobacterium Hyella patelloides
Source: Front Microbiol. 2020 Jul 7;11:1527. doi: 10.3389/fmicb.2020.01527 (PMC7381351; doi:10.3389/fmicb.2020.01527)
Supplement: Supplementary file 1 [file Data_Sheet_1.PDF]

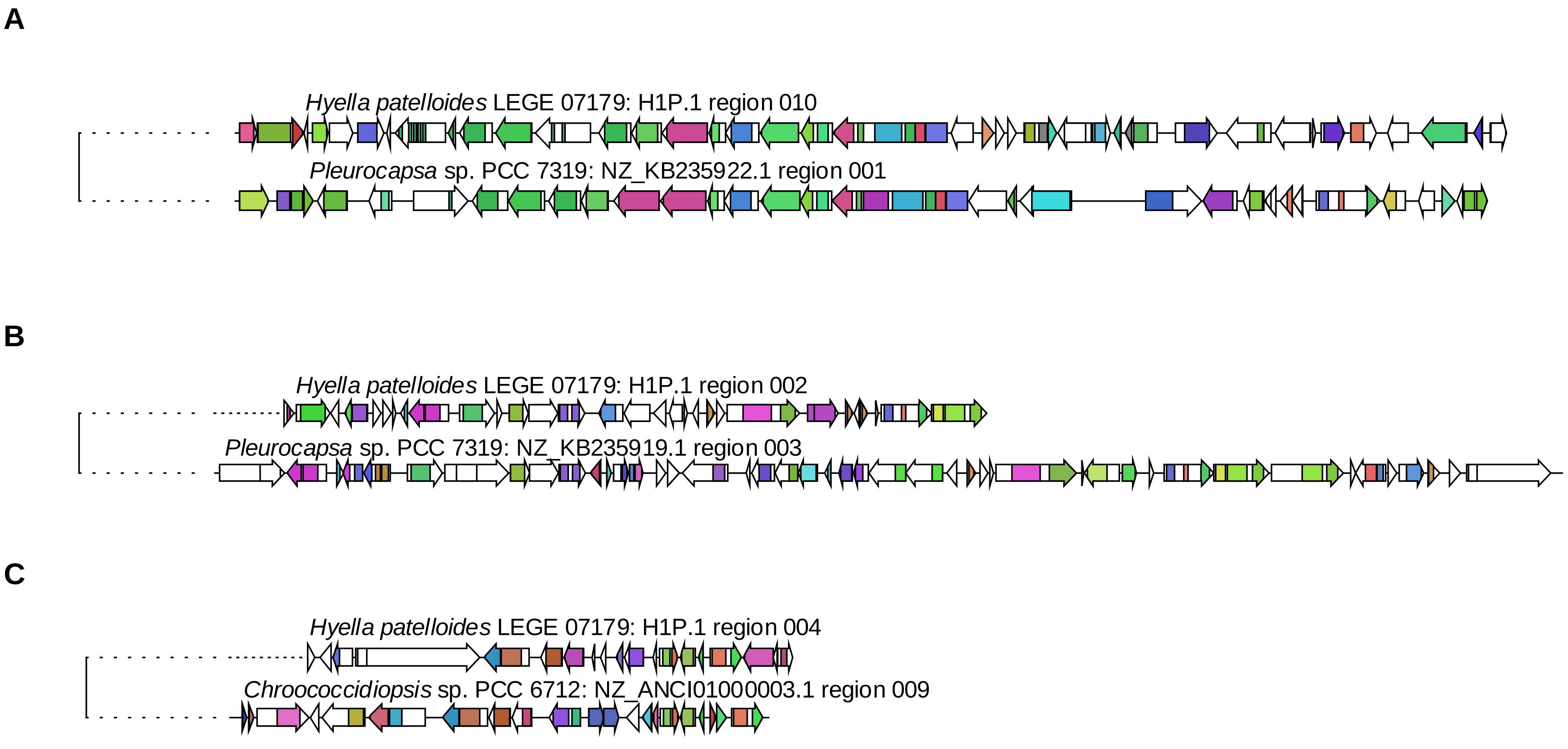

FIGURE S1 - *Hyella patelloides* LEGE 07179 BGCs shared with other baeocyte-forming strain only. **A)** PKS gene cluster shared with *Pleurocapsa* sp. PCC 7319 (Family No 1884); **B)** Bacteriocin/lanthipeptide gene cluster (RiPP) shared with *Pleurocapsa* sp. PCC 7319 (Family No 1882); and **C)** Terpene gene cluster shared with *Chroococcidiopsis* sp. PCC 6712 (Family No 1852). For details see Supplementary Table S14.
