# Supplementary material for: Comparative Genomics Discloses the Uniqueness and the Biosynthetic Potential of the Marine Cyanobacterium Hyella patelloides
Source: Front Microbiol. 2020 Jul 7;11:1527. doi: 10.3389/fmicb.2020.01527 (PMC7381351; doi:10.3389/fmicb.2020.01527)
Supplement: Supplementary file 2 [file Data_Sheet_2.PDF]

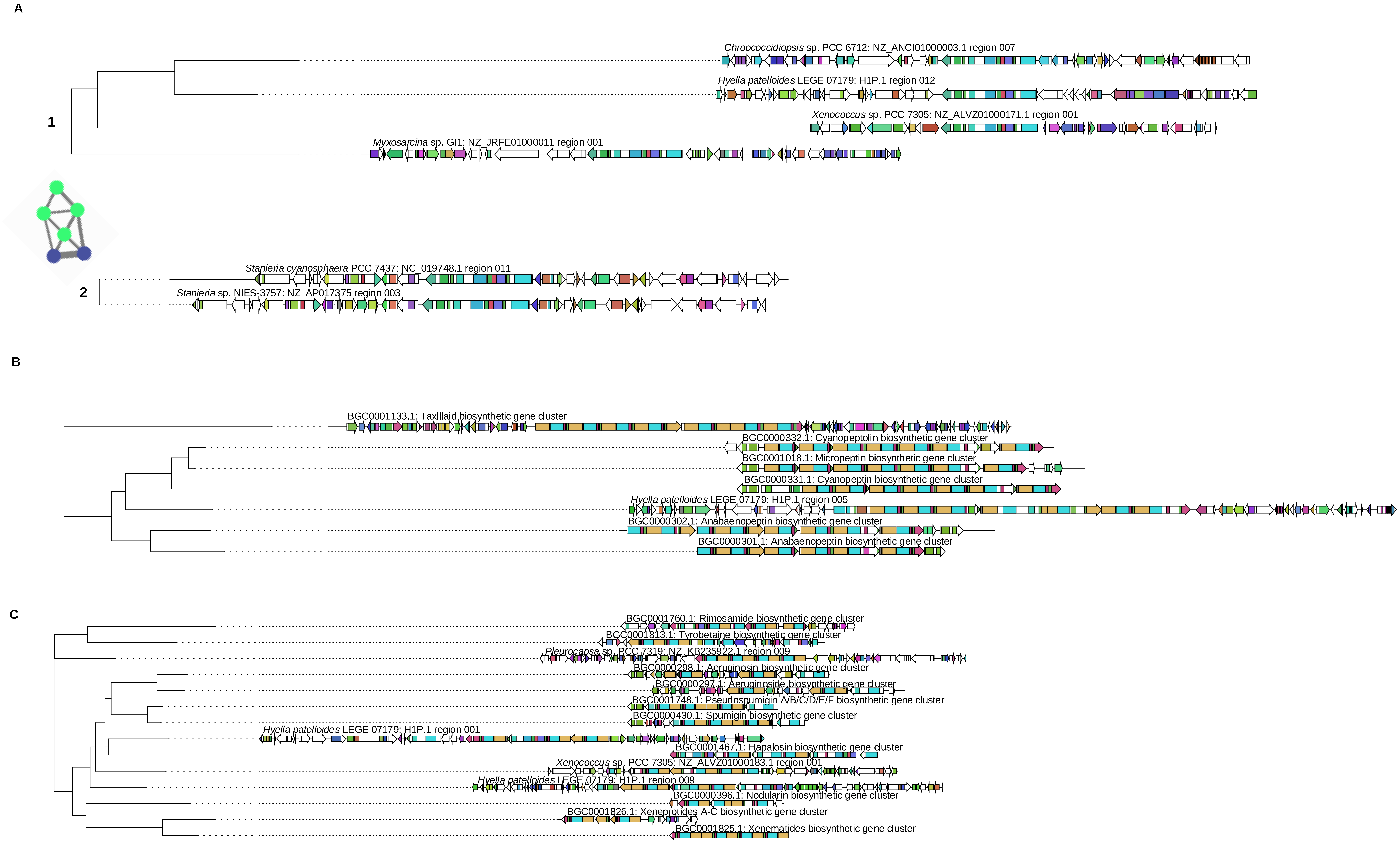

FIGURE S2 - Phylogenetic trees inferring the evolutionary relationships of *Hyella patelloides* LEGE 07179 BGCs shared with other ones from baeocyte-forming strains and MIBiG, within diferent gene cluster families (GCFs). **A)** GCF composed by BGCs (PKS cluster) from *Chroococcidiopsis* sp. PCC 6712, *Xenococcus* sp. PCC 7305 and *Myxosarcina* sp. GI (A1) (Family No 1850) that it is conected with other GCF composed by BGCs (PKS cluster) from *Stanieria cyanosphaera* PCC 7437 and *Stanieria* sp. NIES-3757. (A2) (Family No 1827); **B)** and **C)** GCFs composed by *Hyella patelloides* LEGE 07179 BGC shared with MIBiG BGCs (Family No 1002 and 425, respectively). Phylogentetic trees provided by CORASON (BiG-SCAPE analysis). For details see Supplementary Table S14.
