# Supplementary material for: Comparative Genomics Discloses the Uniqueness and the Biosynthetic Potential of the Marine Cyanobacterium Hyella patelloides
Source: Front Microbiol. 2020 Jul 7;11:1527. doi: 10.3389/fmicb.2020.01527 (PMC7381351; doi:10.3389/fmicb.2020.01527)
Supplement: Supplementary file 3 [file Data_Sheet_3.PDF]

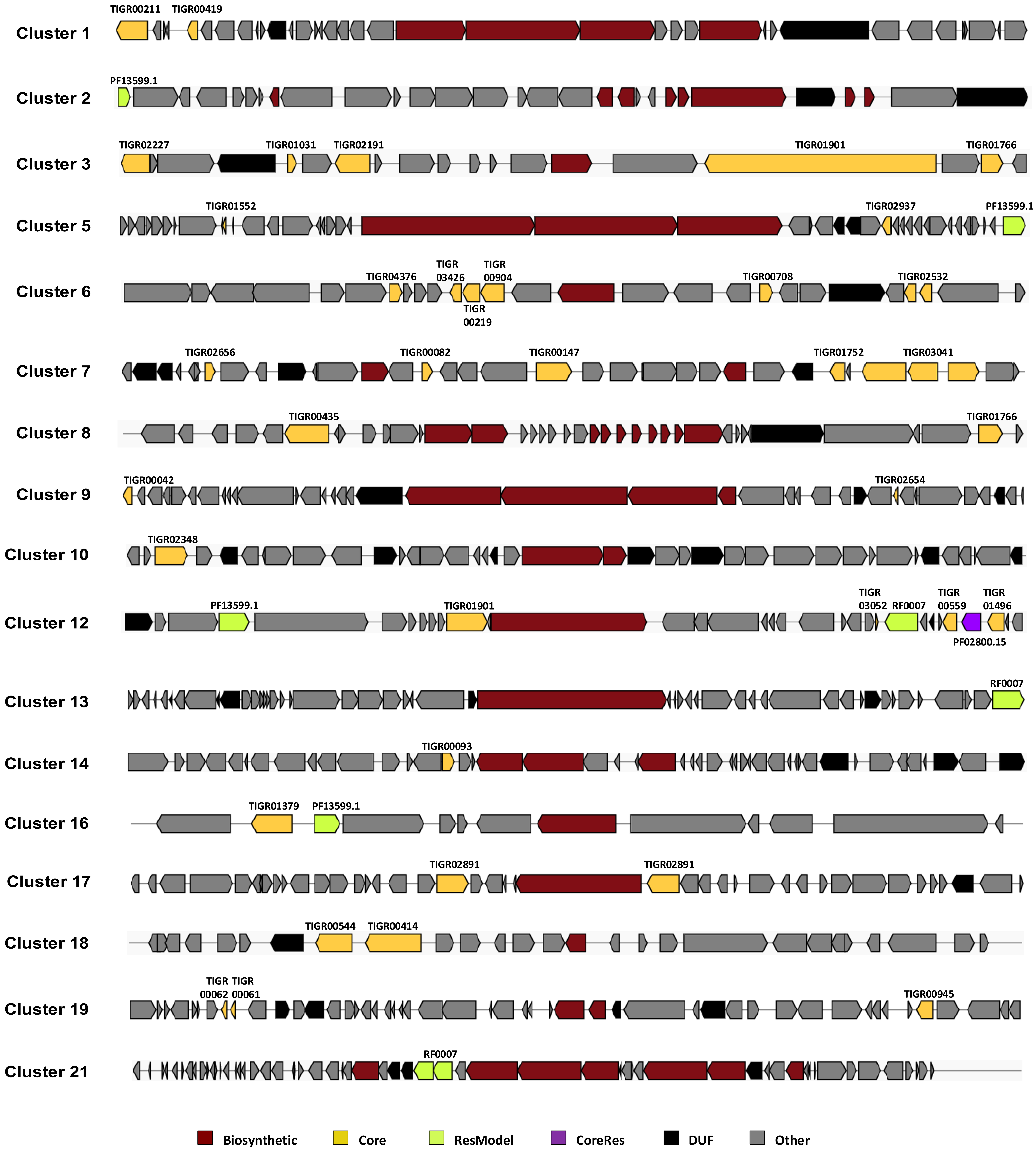

FIGURE S3 - ARTS cluster visualizations showing the localization of the hits identified. The 17 *Hyella patelloides* BGCs that putatively harbour neighboring core and/or resistance genes. DUF – domains of unkonwn function.
