# Supplementary material for: Comparative Genomics Discloses the Uniqueness and the Biosynthetic Potential of the Marine Cyanobacterium Hyella patelloides
Source: Front Microbiol. 2020 Jul 7;11:1527. doi: 10.3389/fmicb.2020.01527 (PMC7381351; doi:10.3389/fmicb.2020.01527)
Supplement: Supplementary file 5 [file Data_Sheet_5.PDF]

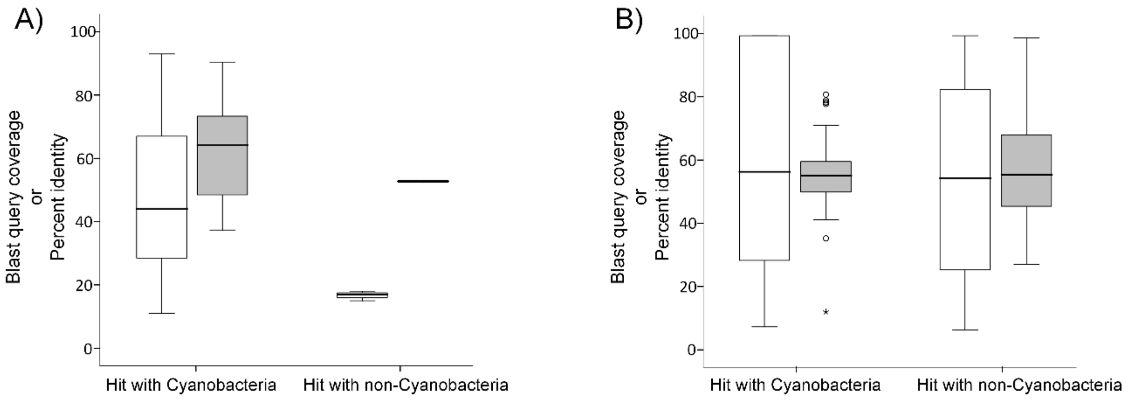

FIGURE S5 - Percentage of the query coverage (white) and identity (grey) of the 20 first hits obtained in the BLASTx analysis using each one of the eleven (A) and twenty-seven (B) contigs obtained in the two genome binning approaches performed.
