# Supplementary material for: Comparative Genomics Discloses the Uniqueness and the Biosynthetic Potential of the Marine Cyanobacterium Hyella patelloides
Source: Front Microbiol. 2020 Jul 7;11:1527. doi: 10.3389/fmicb.2020.01527 (PMC7381351; doi:10.3389/fmicb.2020.01527)
Supplement: Supplementary file 13 [file Data_Sheet_13.PDF]

Supplementary File 1. Fasta file of the 1209 concatenated orthologous genes used in the phylogenetic analyses.

Supplementary File 2. Fasta file of the 126 concatenated genes present in *Hyella patelloides* and *Chroococcidiopsis* sp. PCC 6712 genomes only.

Supplementary File 3. Fasta file of the 62 concatenated genes present in *Hyella patelloides* and *Xenococcus* sp. PCC 7305 genomes only.

Supplementary File 4. Fasta file of the 99 concatenated genes present in *Hyella patelloides* and *Myxosarcina* sp. GI1 genomes only.

Supplementary File 5. Fasta file of 185 genes concatenated present in *Hyella patelloides* and *Pleurocapsa* sp. PCC 7319 genomes only.

Supplementary File 6. Fasta file of the 19 concatenated genes present in *Hyella patelloides* and *Stanieria* sp. NIES 3757 genomes only.

Supplementary File 7. Fasta file of the 26 concatenated genes present in *Hyella patelloides* and *Stanieria cyanosphaera* PCC 7437 genomes only.
