# Supplementary material for: Comparative Genomics Discloses the Uniqueness and the Biosynthetic Potential of the Marine Cyanobacterium Hyella patelloides
Source: Front Microbiol. 2020 Jul 7;11:1527. doi: 10.3389/fmicb.2020.01527 (PMC7381351; doi:10.3389/fmicb.2020.01527)
Supplement: Supplementary file 17 [file Table_4.DOCX]

**Table S4.** Tajima’s relative rate test using a set of 1209 concatenated gene alignments

| **Testing Group** | | **3^rd^ position**^1^ | | | **AA mutations**^1^ | | | |
| --- | --- | --- | --- | --- | --- | --- | --- | --- |
| **(A)** | **(B)** | UA | UB | P value | | UA | UB | P value |
| *H. patelloides* | *Chroococcidiopsis* sp. PCC 6712 | 47795 | 45137 | <0.00001 | | 13708 | 13385 | 0.049 |
|  | *Xenococcus* sp. PCC 7305 | 58890 | 64625 | <0.00001 | | 22382 | 23336 | <0.00001 |
|  | *Myxosarcina* sp. GI1 | 59893 | 67108 | <0.00001 | | 26624 | 26506 | 0.609 |
|  | *Pleurocapsa* sp. PCC 7319 | 60894 | 64893 | <0.00001 | | 26067 | 26051 | 0.944 |
|  | *Stanieria* sp. NIES 3757 | 62670 | 55133 | <0.00001 | | 31144 | 21505 | <0.00001 |
|  | *S. cyanospahera* PCC 7437 | 62549 | 54579 | <0.00001 | | 30956 | 21560 | <0.00001 |
| *Myxosarcina* sp. GI1 | *Pleurocapsa* sp. PCC 7319 | 64343 | 61149 | <0.00001 | | 22594 | 22678 | 0.693 |
| *Stanieria* sp. NIES 3757 | S. *cyanospahera* PCC 7437 | 20046 | 19608 | 0.027 | | 4407 | 4675 | 0.005 |

3^rd^- performed using third codon position only, those that when mutated likely do not change the meaning of the corresponding amino acid.

AA - performed on translated sequences.

UA - Number of unique differences in A.

UB - Number of unique differences in B.

^1^Tajima’s relative rate tests performed using *Cyanothece* sp. PCC 8802 as outgroup.
