# Supplementary material for: Comparative Genomics Discloses the Uniqueness and the Biosynthetic Potential of the Marine Cyanobacterium Hyella patelloides
Source: Front Microbiol. 2020 Jul 7;11:1527. doi: 10.3389/fmicb.2020.01527 (PMC7381351; doi:10.3389/fmicb.2020.01527)
Supplement: Supplementary file 18 [file Table_5.DOCX]

**Table S5.** Number/percentage of genes from species A with a paralog in species B

| **Species A** | **Species B** | **Number (% of all genes in species A)** |
| --- | --- | --- |
| *Hyella patelloides* LEGE 07179 | *Chroococcidiopsis* sp. PCC 76712 | 2256 (27.8%) |
|  | *Stanieria* sp. NIES-3757 | 2236 (27.6%) |
|  | *Myxosarcina* sp. GI1 | 2291 (28.3%) |
|  | *Pleurocapsa* sp. PCC 7319 | 2118 (26.1%) |
|  | *Stanieria cyanosphaera* PCC 7437 | 2303 (28.4%) |
|  | *Xenococcus* sp. PCC 7305 | 2200 (27.1%) |
| *Chroococcidiopsis* sp. PCC 76712 | *Hyella patelloides* LEGE 07179 | 838 (16.2%) |
| *Stanieria* sp. NIES-3757 |  | 898 (18.4%) |
| *Myxosarcina* sp. GI1 |  | 1593 (24.4%) |
| *Pleurocapsa* sp. PCC 7319 |  | 1640 (24.3%) |
| *Stanieria cyanosphaera* PCC 7437 |  | 933 (18.5%) |
| *Xenococcus* sp. PCC 7305 |  | 1090 (20.1%) |
