# Supplementary material for: Comparative Genomics Discloses the Uniqueness and the Biosynthetic Potential of the Marine Cyanobacterium Hyella patelloides
Source: Front Microbiol. 2020 Jul 7;11:1527. doi: 10.3389/fmicb.2020.01527 (PMC7381351; doi:10.3389/fmicb.2020.01527)
Supplement: Supplementary file 20 [file Table_7.DOCX]

**Table S7.** Number/percentage of *Hyella patelloides* LEGE 07179 genes identified as conserved protein of unknown function/protein with recognizable function, transposase or non-conserved protein of unknown function absent in one or more of the baeocyte-forming cyanobacteria analysed.

| **Class^1^** | **CDS total number** | **Conserved protein of unknown function/Protein with recognizable function** | **Transposase** | **Non-conserved protein of unknown function** |
| --- | --- | --- | --- | --- |
| 1 | 681 | 500 (73.4%) | 62 (9.1%) | 119 (17.5%) |
| 2 | 513 | 387 (75.5%) | 28 (5.5%) | 98 (19.0%) |
| 3 | 541 | 351 (64.9%) | 17 (3.1%) | 173 (32.0%) |
| 4 | 582 | 334 (57.4%) | 21 (3.6%) | 227 (39.0%) |
| 5 | 776 | 341 (43.9%) | 13 (1.7%) | 422 (54.4%) |
| 6 | 794 | 178 (22.4%) | 19 (2.4%) | 597 (75.2%) |

^1^Orthologous genes shared between *H*. *patelloides* and five (Class 1; absent in one), four (Class 2; absent in two), three (Class 3; absent in three), two (Class 4; absent in four), one (Class 5; absent in five) strains, and genes only present in *H*. *patelloides* (Class 6; absent in six).
