# Supplementary material for: Comparative Genomics Discloses the Uniqueness and the Biosynthetic Potential of the Marine Cyanobacterium Hyella patelloides
Source: Front Microbiol. 2020 Jul 7;11:1527. doi: 10.3389/fmicb.2020.01527 (PMC7381351; doi:10.3389/fmicb.2020.01527)
Supplement: Supplementary file 22 [file Table_9.DOCX]

**Table S9.** Number of base pairs of the different genomes classified as chromosomal, plasmid or unclassified according to the PlasFlow prediction (using a 0.7 threshold, defaulted parameters).

| **Cyanobacterial strains** | **Chromosome**  **(bp)** | **Plasmid**  **(bp)** | **Unclassified**  **(bp)** |
| --- | --- | --- | --- |
| *Hyella patelloides* LEGE 07179 | 333753 | 2815802 | 4904271 |
| *Chroococcidiopsis* sp. PCC 6712 | 0 | 22813 | 5698074 |
| *Xenococcus* sp. PCC 7305 | 308998 | 829784 | 4790859 |
| *Myxosarcina* sp. GI1 | 520 | 1479757 | 5589582 |
| *Pleurocapsa* sp. PCC 7319 | 0 | 983081 | 6403916 |
| *Stanieria* sp. NIES-3757 | 0 | 143540 | 5319768 |
| *Stanieria cyanosphaera* PCC 7437 | 5943 | 430281 | 5108766 |
